# New Appliances and Things Medical

**Published:** 1906-06-09

**Authors:** 


### New Appliances and Things Medical.

[We shall be glad to receive, at our Office, 28 & 29 Southampton Street, Strand, London, W.C., from the manufacturers, specimens of all new preparations and appliances which may be brought out from time to time.]

### POND'S PATENT TOE-SPRING.

(J. Pond, 21-23 Castle Meadow, Norwich.)

This little appliance is designed for the treatment of hallux valgus. It consists essentially of a steel spring, which encircles the great toe, and a flat plate, which lies against the inner side of the foot. By means of an elastic band, which is passed round the foot, a gentle leverage is exerted on the great toe, tending to reduce any outward displacement which exists. Mankind is very dull and stupid, otherwise it would never have tolerated the tyranny of the modern boot, which condemns everyone alike to have his great toe thrust outwards from its true axis. The result of this is that the foot is weakened, and at the same time subjected to the painful afflictions of ingrowing toe-nail and bunion. Quite a large percentage of elderly people are crippled, or, at any rate, have their activities considerably curtailed, by these miseries; and merely because they have not had the intelligence to revolt against the monstrous boots that habit and fashion have designed. The ancient saying that it is never too late to mend applies as much to our feet as to our morals. And we advise our readers to take an early opportunity of obtaining boots which are straight along the inner side, and of using some simple apparatus, such as the one before us, to wear at night to assist in the process of rectification.

